# Supplementary material for: Native annual forbs decline in California coastal prairies over 15 years despite grazing
Source: PLoS One. 2022 Dec 6;17(12):e0278608. doi: 10.1371/journal.pone.0278608 (PMC9725146; doi:10.1371/journal.pone.0278608)
Supplement: S1 Table — Elevation extracted from the USGS National Map Elevation Point Query Service. Thirty-year mean temperatures and precipitation values extracted from the WorldClim version 2.1 climate model (Fick & Hijmans 2017). (PDF) [file pone.0278608.s001.pdf]

S1 Table. Sites visited by sampling year, ordered from North to South. Elevation extracted from the USGS National Map Elevation Point Query Service. Thirty-year mean temperatures and precipitation values extracted from the WorldClim version 2.1 climate model (Fick & Hijmans 2017).

Site	Approximate Location	2000	2001	2016	2017	Elevation (m)	30 Year Means (1970-2000)	
							Temp. (°C)	Precip. (mm)
Straus	38.18767, -122.89853	×	×	×	×	94	12.90	1034
Inverness Ridge	38.16746, -122.93059	×	×	×	×	125	12.85	1007
Bull Point	38.08140, -122.95979	×	×	×	×	17	12.80	962
Chimney Rock	37.99430, -122.98326	×	×	×	×	93	12.68	961
Wildcat Park	37.92099, -122.26422	×	×	×	×	349	14.00	767
Pomponio	37.28853, -122.40416	×	×	×	×	49	12.70	712
Laguna Creek	37.01304, -122.13373	×	×		×	248	13.20	847
Wilder Creek	36.98177, -122.06415	×	×	×	×	124	13.51	777
Porter Ranch	36.86937, -121.74267	×	×	×	×	31	13.90	490
Point Lobos	36.52499, -121.92256		×	×	×	23	13.03	517
