# Supplementary material for: Native annual forbs decline in California coastal prairies over 15 years despite grazing
Source: PLoS One. 2022 Dec 6;17(12):e0278608. doi: 10.1371/journal.pone.0278608 (PMC9725146; doi:10.1371/journal.pone.0278608)
Supplement: S2 Table — Nomenclature follows Jepson Flora Project (2020). (PDF) [file pone.0278608.s002.pdf]

S2 Table. Native annual forb species observed in sampling periods 1 and 2. Nomenclature follows Jepson Flora Project (2020).

Species Name	Present in Sample 1	Present in Sample 2
Acmispon americanus	x	x
Acmispon brachycarpus		x
Acmispon parviflorus	x	
Acmispon strigosus	x	x
Acmispon wrangelianus	x	x
Aphanes occidentalis	x	
Apiastrum angustifolium	x	x
Calandrinia menziesii	x	x
Cardamine oligosperma	x	
Castilleja ambigua		x
Castilleja densiflora	x	x
Castilleja floribunda	x	
Cicendia quadrangularis	x	
Clarkia davyi	x	x
Claytonia perfoliata	x	x
Crassula connata	x	
Cryptantha sp.	x	
Daucus pusillus	x	x
Deinandra corymbosa	x	x
Epilobium species	x	
Hesperevax acaulis	x	
Hesperevax sparsiflora	x	
Lasthenia californica	x	x
Layia platyglossa	x	x
Lepidium nitidum	x	x
Leptosiphon parviflorus	x	
Lupinus species	x	
Lupinus bicolor	x	x
Lupinus hirsutissimus	x	
Lupinus nanus	x	x
Lysimachia minima	x	x
Madia sativa	x	x
Micropus californicus	x	
Microseris douglasii	x	
Montia fontana	x	

Plagiobothrys chorisianus	x	x
Plagiobothrys trachycarpus	x	x
Plantago erecta	x	x
Psilocarphus brevissimus	x	
Psilocarphus tenellus	x	
Pterostegia drymarioides	x	
Trifolium albopurpureum	x	x
Trifolium barbigerum	x	x
Trifolium bifidum	x	
Trifolium depauperatum	x	
Trifolium gracilentum	x	
Trifolium macraei	x	x
Trifolium microdon	x	x
Trifolium trichocalyx	x	
Trifolium variegatum	x	
Trifolium willdenovii	x	
Triphysaria floribunda	x	
Triphysaria pusilla	x	x
Triphysaria versicolor	x	x
Zeltnera davyi	x	
Zeltnera muehlenbergii	x	
