# Supplementary material for: Native annual forbs decline in California coastal prairies over 15 years despite grazing
Source: PLoS One. 2022 Dec 6;17(12):e0278608. doi: 10.1371/journal.pone.0278608 (PMC9725146; doi:10.1371/journal.pone.0278608)
Supplement: Attachment — Submitted filename: Review.docx [file pone.0278608.s003.docx]

Authors examined the effects of livestock grazing by cattle on grassland diversity and cover in California. This is a timely topic and adapting grazing management to account for changing climate will be critical for conserving rangelands worldwide. The authors are lacking key pieces of information in the methods section, and the discussion section must draw conclusions more tightly linked to actual findings, and contextualize findings in the context of current literature.

Introduction

Page 3, Line 25: I’m not familiar with the term semi-natural plant community, define?

Page 3, Line 38: Is grazing only beneficial in highly invaded grasslands?

Materials and methods

Page 5: no need to include ‘the two of us’

Page 5: Please provide more information about the grazed and ungrazed plots. At what intensity (AUM) were the plots grazed? What size are the plots? When surveying the vegetation did you avoid the fenceline / edge effects? At what frequency were the plots grazed? How close were the paired sites (are they bisected fields?)? Please discuss herbivory within both grazed and ungrazed plots by wild ungulates.

Page 5: Site descriptions. No need to talk about survey permission, just describe the sites that you actually included in the study. Could you include a map of these study areas and/or a description of the range of conditions across which these plots occur (distance apart, elevational range, etc.). In other words, tailor site description to the actual study sites.

I see here that it was hard to establish a complete grazing history, but could you provide a ball park estimate of grazing intensity in order to contextualize that patterns that you observe with other studies?

Line 99: It is going to be hard to attribute differences in response to grazing to climate (especially with incomplete grazing history), with only two time points (though I agree it is likely). We two sample points, however, many other factors could have changed – for instance, land use in the surrounding habitat matrix, nitrogen deposition, pressure from undomesticated livestock. In other words, attributing differences to drought specifically is challenging, and should be discussed in the discussion.

Line 106: Does this sampling regime recapitulate the original veg sampling?

Line 125: Just the version of R is fine – though I do like ‘Bird Hippie’.

Line 128: How is year different from time period – somehow I didn’t understand that you had multiple years of data within each time period – could you make this more clear?

Results

In terms of vegetation height, did you derive this via the trait database or did you collect measurements in the field, and if the latter, were measurements collected after the fields had been grazed? Sorry, that is more of a methods question!

Could you provide more information about the overall community composition – in particular, I’m curious about invasive species cover, perennial grass cover, etc.

Discussion

Line 185: As mentioned, I need a little more information before I can contextualize this height difference.

Line 214: Can you connect your findings here with other studies of grazing in similar habitats?

Line 227: Since your statistical tests indicate no differences, do not discuss as if there are treatment effects.

Line 222: Did changes occur due to composition change or due to increased biomass?

Line 245: This is good – could mention other forms of change in the region that could explain differences, but supporting your finding with the mesic index etc is good.

Line 252: Be clear here about what grazing effects (positive effects on diversity?). Several studies have shown that negative grazing impacts on diversity and native cover are exacerbated during drought years.

Live 253: This transition to annual dominated states has been observed in other studies – maybe cite a few?

Live 276: I don’t feel like you’ve set us up to make conclusions about microhabitat (did you do any spatial studies?) or to mention assisted migration. In order to mention these things, please explain how your findings support this suggestion.
