# Supplementary material for: Native annual forbs decline in California coastal prairies over 15 years despite grazing
Source: PLoS One. 2022 Dec 6;17(12):e0278608. doi: 10.1371/journal.pone.0278608 (PMC9725146; doi:10.1371/journal.pone.0278608)
Supplement: Attachment — Submitted filename: Responses to Reviewers.docx [file pone.0278608.s004.docx]

**Responses to reviewer comments**

Reviewer 1: Authors examined the effects of livestock grazing by cattle on grassland diversity and cover in California. This is a timely topic and adapting grazing management to account for changing climate will be critical for conserving rangelands worldwide. The authors are lacking key pieces of information in the methods section, and the discussion section must draw conclusions more tightly linked to actual findings, and contextualize findings in the context of current literature.

Introduction

Page 3, Line 25: I’m not familiar with the term semi-natural plant community, define?

*We agree this was unclear and realized that we did not need this term to make our main point in this sentence, so we have removed it.*

Page 3, Line 38: Is grazing only beneficial in highly invaded grasslands?

*We have modified our language here to better reflect our meaning: grazing is beneficial in this system, where invasive annual grasses grow rapidly and can outcompete native species.*

Materials and methods

Page 5: no need to include ‘the two of us’

*We have removed this phrase.*

Page 5: Please provide more information about the grazed and ungrazed plots. At what intensity (AUM) were the plots grazed? What size are the plots? When surveying the vegetation did you avoid the fenceline / edge effects? At what frequency were the plots grazed? How close were the paired sites (are they bisected fields?)? Please discuss herbivory within both grazed and ungrazed plots by wild ungulates.

*We have added details about the size of pastures (plots), setback from edge effects, proximity of paired plots, and wild ungulates at the sites in the site description. As we explain, we tried to collect data on grazing intensity and timing from land management reports and conversations with landowners, but the information was sparse and general (e.g. general descriptions of the type of operation rather than actual intensity of grazing in the specific years of our study). So, we describe the typical grazing regime in our sites and region.*

Page 5: Site descriptions. No need to talk about survey permission, just describe the sites that you actually included in the study. Could you include a map of these study areas and/or a description of the range of conditions across which these plots occur (distance apart, elevational range, etc.). In other words, tailor site description to the actual study sites.

*We included the information about survey permission, per the PLOS One guidelines to authors. We would be willing to remove it if the editor prefers but have left it in for now. We have added elevation and 30-year historical mean temperature and annual precipitation data to Table S1 in order to help the reader understand variation in the sites. A map of the sites is available in Table S1 as well. We have also added some language to page 5 to describe the sites and how far apart the plots are.*

I see here that it was hard to establish a complete grazing history, but could you provide a ball park estimate of grazing intensity in order to contextualize that patterns that you observe with other studies?

*We gave added general information about grazing intensity and duration as reported by ranchers and land managers in the region.*

Line 99: It is going to be hard to attribute differences in response to grazing to climate (especially with incomplete grazing history), with only two time points (though I agree it is likely). With two sample points, however, many other factors could have changed – for instance, land use in the surrounding habitat matrix, nitrogen deposition, pressure from undomesticated livestock. In other words, attributing differences to drought specifically is challenging, and should be discussed in the discussion.

*We agree with the reviewer that we cannot conclusively determine the cause of the decline in native annual forbs with data from two time points in light of many likely co-occurring changes in the landscape. Therefore, throughout the discussion, we acknowledge this challenge: in lines 251-254 and 282-284 we clearly state that our hypotheses are supported by circumstantial evidence and that we do not have experimental data to support our conclusions.*

Line 106: Does this sampling regime recapitulate the original veg sampling?

*Yes – the methods used in the second time period are the same as in the original sampling. We’ve added language early in the methods section to clarify this point.*

Line 125: Just the version of R is fine – though I do like ‘Bird Hippie’.

*We’ve removed the name of the R version from the text.*

Line 128: How is year different from time period – somehow I didn’t understand that you had multiple years of data within each time period – could you make this more clear?

*We’ve added language explaining that each sampling period consisted of sampling in two subsequent years to incorporate some interannual variation.*

Results

In terms of vegetation height, did you derive this via the trait database or did you collect measurements in the field, and if the latter, were measurements collected after the fields had been grazed? Sorry, that is more of a methods question!

*Specific leaf area data were gathered from the TRY Trait Database as well as field sampling. This is explained in the methods. When we were able to supplement database traits with traits we collected in the field, they usually came from grazed areas, as many of the native annual forbs were not present in the ungrazed plots. We collected leaves from individuals that showed now evidence of recent grazing. We’ve added language to the methods to clarify these points.*

Could you provide more information about the overall community composition – in particular, I’m curious about invasive species cover, perennial grass cover, etc.

*Our manuscript focuses on changes in the richness and cover of native annual forbs across several grazed prairies over time, and we provide circumstantial evidence to evaluate hypotheses of which factors have driven this trend. Whereas we agree that other community composition changes are interesting, the sampling protocol was designed to answer our primary questions about the changing effects of grazing on native annual forbs, and we did limited sampling of the other guilds. So, we have chosen to stay focused on our primary questions about declines in native annual forbs.*

Discussion

Line 185: As mentioned, I need a little more information before I can contextualize this height difference.

*We have added the values to the text to help the reader visualize the differences, and described that how the heights compare to one another over time.*

Line 214: Can you connect your findings here with other studies of grazing in similar habitats?

*We had already cited a meta-analysis showing that grazing has positive effects on native annual forbs in California grasslands. We have added references to similar results from other Mediterranean grasslands globally, including a global review that shows that grazing favors annual and short-stature species.*

Line 227: Since your statistical tests indicate no differences, do not discuss as if there are treatment effects.

*As illustrated in Table 1, there were significant differences in shrub cover. We have reworded this sentence to be clearer that there was an interactive effect of time period and grazing treatment since the difference in the shrub cover over time for ungrazed plots was much greater than in grazed plots.*

Line 222: Did changes occur due to composition change or due to increased biomass?

*We are not sure that we fully understand this comment which refers to a sentence on former lines 221-223. We think the reviewer is asking for the mechanism by which a lower canopy cover would favor annual forbs. We have modified the sentence to add the underlined text. “The significantly taller vegetation height in the second sampling period may explain part of the decline in native annual forb richness, given that many of the native annual forbs in California are low-stature species that benefit from greater light availability afforded by a shorter canopy.”*

Line 245: This is good – could mention other forms of change in the region that could explain differences, but supporting your finding with the mesic index etc is good.

*We are glad that the reviewer finds our mesic index to be useful. We have thoroughly discussed what we consider to be the most plausible factors driving the patterns we observed. There are always more potential factors that could be discussed, but without specific suggestions for alternative mechanisms that we should consider.*

Line 252: Be clear here about what grazing effects (positive effects on diversity?). Several studies have shown that negative grazing impacts on diversity and native cover are exacerbated during drought years.

*We agree with the reviewer that this sentence was somewhat misleading and have removed it, since we discuss patterns of native annual forb cover in the following paragraph.*

Live 253: This transition to annual dominated states has been observed in other studies – maybe cite a few?

*We didn’t fully understand this comment. We are discussing differences in annual forb cover across years but are not suggesting that the system will transition that the system is transitioning from a perennial to an annual dominated system, at least not during the time period of our study. In fact, the system has long been dominated by non-native annual grasses.*

Live 276: I don’t feel like you’ve set us up to make conclusions about microhabitat (did you do any spatial studies?) or to mention assisted migration. In order to mention these things, please explain how your findings support this suggestion.

*Our results suggest a decline in species adapted to mesic conditions with increasing aridity, which raises the questions of how to conserve these species. We have toned down our language to clarify that our results suggest that land managers should consider a range of potential actions to protect the mesic-condition species that were lost, rather than us promoting specific management actions.*

Reviewer #2: Review of PLOS

This is an excellent paper that resamples a coastal prairies to explore how a regional drought influenced species richness. The sampling approach was straightforward, as was the data analysis. The results were unambiguous and certainly worthy of publication. The writing was clear and concise and addressed the key limitations and strengths of the analysis. Overall, a very clean manuscript. *Thank you.*

There are a few areas where I believe there are opportunities for improvement or increased impact for this publication. Here are my suggestions:

1. Given the broad regional sampling that was conducted, there may be opportunities to get a more nuanced view of the impact of climate change/drought. It is highly unlikely that the extent of the drought (deviation from mean conditions) or the 2017 pulse in increased precipitation was equal across the entire gradient. I would appreciate seeing the nuance of species loss or increase in vegetation height relative to the extent of drought/2017 pulse across the gradient rather than a single, aggregated measure.

*We agree with Reviewer #2 that adding nuance to understand how variation in precipitation or temperature changes would improve our interpretation of the results. However, not all of our sites have nearby weather stations, so the trends we do present are aggregated from available weather data at several stations in the region. Hence we are wary of using interpolated weather data for such an analysis.*

2. Somewhere either in the supplementary materials or in a table in the paper there should be a list of species, their abundance, and an identification of which species were lost. This would be a valuable resource.

*We have added a supplementary table listing all species present and lost in both time periods (Table S2).*

A few other minor edits.

Line 40: Provide spatial description for readers not familiar with California geography; consider including biophysical data that capture the environmental gradient.

*We have added information about the elevation, mean temperature, and annual precipitation to Table S1 to help the reader understand the biophysical factors at our sites. Additionally, a map of sites is available in fig. S1.*

Line 53: Global patterns don’t help in interpreting these data; need to include weather trends and key disruptive weather events that are useful in understanding the changes observed.

*We have modified the sentence to focus on California and added two California-specific references.*

Line 98: More precision here. Much of the explanation hangs on a single source describing an “exceptional drought”. I want to see the actual deviation, including the temporal extent of dry periods, the relative change in water year, increases in mean temperature, etc. for the sampled region.

*Unfortunately, as discussed previously most of our field sites do not have weather stations. However, we cite two studies which describe the severity and unusual conditions of the drought throughout California: Swain et al. 2014 and Griffin and Anchukaitis 2014. Furthermore, we have added study-period mean lines to figure S1 to provide context for the region, using the met station data that are available to us.*

Line 195: Shrub encroachment as a topic comes out of nowhere. It hasn’t been established as a key metric nor are data presented. I’d drop this argument or add the woody plant data.

*We mostly disagree with Reviewer #2 here: in the prior version we introduced woody shrub encroachment in line 32-33, and described our collection and analysis of shrub data in lines 112-113 and 125-131. We described the changes in shrub cover in lines 146-148, and interpreted these results in lines 195 to 201. However, we have introduced the term encroachment at the end of the first paragraph of the introduction to ensure that our terminology is clear, which we think may have been the confusion.*

Line 221: The most parsimonious explanation is that a higher-than-average water year produced greater plant biomass in this water-limited ecosystem.

*We feel that that the taller vegetation is likely due to a combination of increased precipitation (in 2017 at least; 2016 was a relatively average year), as suggested by the reviewer, and decreased grazing pressure as inferred from conversations with landowners.*

Line 237: Unclear what “Climatic Water Deficit” is as a proper noun. First time it is introduced and it isn’t well explained.

*We have changed the wording of this line to more clearly refer to plant-available water as opposed to the undefined “Climatic Water Deficit”.*

Overall, I believe this paper to be valuable to understanding the factorial influence of grazing and weather on forb species richness in a biodiverse ecosystem.

*We appreciate the positive feedback.*
