# Supplementary material for: Native annual forbs decline in California coastal prairies over 15 years despite grazing
Source: PLoS One. 2022 Dec 6;17(12):e0278608. doi: 10.1371/journal.pone.0278608 (PMC9725146; doi:10.1371/journal.pone.0278608)
Supplement: Attachment — Submitted filename: GrazingResurveyManuscript_PLOS One_revised-resubmission.docx [file pone.0278608.s005.docx]

**Title:** Native annual forbs decline in California coastal prairies over 15 years despite grazing

**Short title**: Changes in effect of grazing on coastal prairies over 15 years

**Authors and addresses:**

Josephine C. Lesage^1,3^, Grey F. Hayes^2^, Karen D. Holl^1*^

^1^ Environmental Studies Department, University of California, Santa Cruz, Santa Cruz, CA 95064

^2^ Swanton Pacific Ranch, Cal Poly San Luis Obispo, Davenport, CA 95017

^3^ Santa Barbara Botanic Garden, 1212 Mission Canyon Rd, Santa Barbara, 93105

*Correspondence:

Karen D. Holl, Environmental Studies Department, University of California, Santa Cruz, Santa Cruz, CA 95064, kholl@ucsc.edu

**Abstract**

Livestock grazing is often used as a land management tool to maximize vegetation diversity in grassland ecosystems worldwide. Prior research has shown that cattle grazing benefits native annual forb species in California’s coastal prairies, but drought and increasing aridity may alter this relationship. In 2016 and 2017, we resurveyed the vegetation structure, native annual forb cover, and native annual forb richness in ten grazed and ungrazed prairies that were originally measured in 2000 and 2001 along a 200-km gradient from Monterey to Sonoma counties in California. We found that grazed prairies continued to have significantly lower vegetation height and thatch depth than ungrazed prairies, and that shrub encroachment over the 15-year period was significantly greater in ungrazed prairies. Furthermore, grazed prairies continued to have greater native annual forb richness (4.9 species per site) than ungrazed sites (3.0 species per site), but native annual forb richness declined by 2.8 species per site in grazed prairies and 0.1 species per site in ungrazed prairies between survey periods. We suggest that severe drought and increasing aridity may be driving declines in native annual forb richness in grazed prairies. The species we recorded only in earlier surveys were disproportionately wetland-associated and had higher average specific leaf area than species that remained through the second survey period. Finally, the cover of native annual species increased regardless of whether prairies were grazed, suggesting that the high precipitation in 2017 may have benefitted the native annual forb species that persisted at sites between surveys. Our study shows that weather conditions affect the outcomes of land management strategies.

**Key words:** grassland, land management, grazing, drought, shrub encroachment, climate change

**Introduction**

Grazing affects nearly half of the Earth’s savanna, grassland, and steppe biomes [1], and human activities often alter the diversity of plant communities [2, 3]. Inappropriate grazing regimes can result in widespread ecosystem degradation and diversity loss, especially in arid and semi-arid environments [4-6]. Well-managed livestock grazing, however, can benefit native vegetation diversity and cover in grass-dominated ecosystems [7-10]. At moderate stocking rates in mesic grasslands, large mammal grazing increases the floral diversity of South American steppe [11], Mediterranean-climate grasslands [10, 12], midwestern United States tallgrass prairies [13], and northern European semi-natural grasslands [14]. Furthermore, livestock grazing can reduce the spread of woody shrubs and trees into grasslands, precluding successional conversion to shrubland or forest in the absence of disturbance [15-18], also known as shrub or forest encroachment.

In Mediterranean climates, grazing generally benefits short-statured species [19-21], though the strength and direction of this effect can vary depending on annual precipitation [22-24]. In systems like California coastal prairies, where non-native vegetation grows quickly and can limit native species germination and growth, grazing in grasslands has been promoted as a conservation strategy to enhance the diversity of native species [19, 25], as it serves to reduce the cover, biomass, and litter of fast-growing, tall-statured exotic annual grasses.

We focus on California’s coastal prairies, a highly diverse grassland type. These grasslands are present from Santa Barbara county into Oregon, where winter precipitation is relatively high and coastal fog alleviates summer drought [26]. Among North American grassland ecosystems, coastal prairies are exceptional for their high native species richness and they have higher native species cover than most other grassland types in California [27]. Native annual forbs represent between 25-60% of the recorded species in California’s coastal prairies, although they often do not contribute greatly to vegetation cover [19, 26-28].

In 2000 and 2001, Hayes and Holl (19) found strong evidence that moderate levels of cattle grazing favored small-statured native annual forbs in California coastal prairies. In 2016 and 2017, we sought to answer whether such grazing continues to benefit native annual forbs in California’s coastal prairies, particularly given the extreme drought and temperature conditions in the years preceding the study. California is already experiencing, 1) increasingly ‘extreme’ precipitation, wherein rainfall events occur less frequently, but in larger magnitudes; 2) increasing ‘whiplash’ weather, when exceptionally dry and wet periods follow one another with minimal change in net precipitation; and 3) rising temperatures [29-31]. Over the last few decades, California grasslands have experienced increasing temperatures and aridity [32] and a 1-in-1200-year drought from 2012-2014 [33]. Both of these climatic anomalies have impacted the species composition of California grasslands, leading to declines of some drought-sensitive species, particularly native annual forbs [34, 35].

We hypothesized that native annual forb richness and cover had declined in these grasslands since 2001 due to the recent severe drought and overall increasing aridity, but that declines in richness and cover would be less severe in grazed grasslands because grazing reduces the competitive dominance of shrubs. To evaluate whether potential declines were related to changes in weather and/or changes in the surrounding vegetation structure, we analyzed climate-relevant traits of the native annual forb species in our sites and changes in vegetation structure over time. We compared the wetland indicator status of species that were recorded in the earlier but not later surveys, which should reflect whether those species were more adapted to mesic environments. We also examined species’ specific leaf area (SLA), which is often associated with drought tolerance [35-37]. We hypothesized that the native annual forb species missing in the later survey would largely be adapted to moister conditions.

Results mainly show forb and shrub data, so reviewing the forb, grasses, shrubs, and other concepts used in the manuscript is crucial because it is confusing. Please, standardize the main concepts used in the manuscript.

**Materials and methods**

*Site descriptions*

In 2000 and 2001, we surveyed 26 paired grazed and ungrazed coastal prairie sites across 400 km of coastline between San Luis Obispo and Mendocino counties in California, USA [19]. In 2016 and 2017, we resampled sites along 200 km of coastline, in the central area of the prior survey extent (Monterey to Sonoma Counties) where we could obtain permission from landowners for access and grazing patterns had been maintained since 2000-2001 (S 1 Figure; S1 Table). All analyses presented in this paper include only data from these ten sites. For simplicity, we refer to the 2000-2001 sampling period as Time 1 (T1) and the 2016-2017 sampling period at Time 2 (T2). Thus, each sampling period represents two subsequent years of sampling and all but two sites were sampled four times (S1 Table).

Please, delete the Year categories in "Vegetation Height" and the "Thatch Depth" figures, keeping only those categories in the Shrubs cover Figure; also, put the letter "c" on the "Cover" word in capital.

At all sites, paired plots are > 2 km apart, on similar slopes and aspects. Sites range from 1-20 ha; transects within sites were separated by 15 m to 1 km and placed at least 5 m from fence lines to avoid edge effects. Sites range from 20 m to 350 m in elevation, and have 1970-2000 30-year temperature and precipitation means within 1.3°C and 544 mm of one another based on interpolated climate data [S1 Table; 38]. There was no evidence of prior mechanical soil disturbance, and soils were deeper than 40 cm and not ultramafic (serpentine).

Fires in California are worldwide famous. Please indicate that study sites were not affected by fires because, in my opinion, they are a factor to consider in this study (but I can be wrong).

We compiled available grazing regime information from landowners and agency reports, but in many cases, specific data on the intensity and timing of grazing for our sites were not available. Cattle were the only domestic grazing animals at our sites. The typical grazing regime in the region is at 1 cow per 2-4 ha with grazing occurring year-round at most sites by cow-calf or dairying operations. Based on our conversations with landowners, grazing pressure at T2 was maintained at or slightly below T1 levels. The only wild ungulate at these sites is the Colombian black tailed mule deer (*Odocoileus hemionus columbianus*); we did not find evidence of grazing by this species, but access is not restricted at our sites. Deer primarily eat broadleaved plants and cattle primarily graminoids [39]. Moreover, a recent study suggests that wild ungulate grazing in California has minor impacts on plant communities relative to cattle grazing [40].

(0.5 ind ha^−1^ yr^−1^)???

Please add information about the cattle`s type and a reference of its weight, for example, Bos taurus ~300 kg.

(1.0 ind ha^−1^ yr^−1^)???

*Climate and weather data*

California’s Mediterranean ecosystems have high interannual variability in rainfall quantity and timing, though all precipitation generally falls between October and April and rainfall between May and September is rare. We gathered all precipitation and temperature information available from weather stations in Santa Cruz, Monterey, San Francisco, and Marin counties from NOAA’s Climate Data Online archive (<https://www.ncdc.noaa.gov/cdo-web/>). Precipitation in the 2000 water year (October 1, 1999 to September 30, 2000) was near the 100-year average (449 to 1328 mm) and in 2001 was below average (416 to 912 mm) at our study sites. Precipitation was close to average in the 2016 water year (511 to 1085 mm) and substantially greater than average in 2017 (673 to 1958 mm). Growing season precipitation (November-April) followed similar trends, and temperatures were similar in both sets of sampling years (S2 Fig). However, the second set of sampling years occurred immediately after an exceptional 1-in-1200 year drought [33, 41], which was accompanied by above-average temperatures (S2 Fig).

*Vegetation structure and native annual forbs*

Data collection replicated methods used by Hayes and Holl (2003) and are described further below. We sampled from April through May, traveling from southern sites to northern sites to follow the peak flowering phenology of most species. At each site, we relocated T1 50-m line transects using a Garmin eTrex 20 GPS. There were five transects each in the grazed and ungrazed portions at each site.

We quantified community composition by recording the identity of each species that intersected a 1.8-mm-diameter pin at 1-m intervals. We recorded each species that intercepted the pin once. Our nomenclature and species origin information follow the Jepson Manual and online Jepson eFlora database [42]. We measured vegetation height at 5-m intervals using a piece of paper dropped onto foliage and recording the lowest point, and thatch depth (*i.e.,* build-up of dead plant biomass) by pushing a 1.8-mm pin to the soil surface and measuring the tallest standing dead biomass. We calculated shrub cover as the number of woody shrubs intercepts along each transect as a percent of the 50 intercepts total along the transect. Finally, we carefully searched for and quantified the cover of all native annual forbs (to the nearest cm^2^) present within a 1-m belt transect centered over the 50-m transect.

We compiled specific leaf area (SLA, leaf area/leaf dry mass) for 43 of the 56 native annual forbs species from the TRY Plant Trait Database and data collected by others [35, 43, 44]. We also collected data from species present in and near our sites during spring and summer 2018. Most of these data came from grazed plots, since this is where the species were more prevalent, though we selected individuals that showed no evidence of recent grazing. When data for a single species was available from multiple sources, we averaged the values. High SLA is associated with drought-intolerance and low water use efficiency [45, 46]. We used the United States Army Corps of Engineers National Wetland Plant List to determine the wetland indicator status of all native annual forbs found in our sites [47].

*Data analysis*

We conducted all data analyses in R version 4.1.2 [48]. To model the effects of time and grazing treatment on vegetation structure (*i.e.,* vegetation height, thatch depth, and shrub cover) and native annual forbs (*i.e.,* richness and cover), we used generalized linear mixed models in the glmmTMB package [49]. We treated sampling period and grazing treatment (both categorical with two levels) as fixed effects and treated year and transect as nested within-site random factors. We fit vegetation and thatch height with normal distributions, shrub cover using a beta distribution, and native richness using a Poisson distribution. Cover data were fit using a Tweedie distribution because our data were overdispersed and zero-inflated. Model fit was checked using the DHARMa package to plot residuals against fitted values [50].

We summarized the SLA of native annual forb species for the sampling period when they were found (*i.e.:* lost, observed in only T1; still present, observed in both T1 and T2; and new, observed in only T2) and compared the values using one-way ANOVA followed by Tukey Honest Significant Difference tests (*p* < 0.05). We also compared the US Army Corps of Engineer wetland indicator status of the native annual forbs found during T1 and T2 to determine whether those in T2 were less likely to be wetland-associated.

I do not believe these data analyses and results were subsequently discussed in the manuscript. Is this analysis necessary or valuable for the paper?

Results

Vegetation height was greater in both ungrazed and grazed prairies in the second sampling period than in the first, and, as expected, vegetation was consistently shorter in grazed than ungrazed prairies across both periods (Fig 1; Table 1). Grazed prairies had lower thatch depth than ungrazed prairies across all sampling years, and thatch depth did not change significantly over time (Fig 1; Table 1). Woody shrub cover, primarily of the native species *Baccharis pilularis*, *Rubus ursinus*, and *Toxicodendron diversilobum,* increased significantly more in ungrazed prairies than in grazed prairies between the two sampling periods.

Please cite the figure or table that shows these results.

**Fig 1.** Vegetation height, thatch depth, and shrub cover over time in grazed and ungrazed prairies. Values represent means for n = 10 sites and error bar indicate 1 SE.
**Table 1.** Generalized linear model results for changes in vegetation factors across treatments and over time.

| **Model** | **Parameter** | **Estimate** | **Std. Error** | **z-value** | **p-value** |
| --- | --- | --- | --- | --- | --- |
| Vegetation height | Intercept | 25.87 | 2.44 | 10.60 | <0.001 |
|  | Sampling Period | 10.43 | 1.93 | 5.42 | <0.001 |
|  | Treatment | -11.24 | 1.56 | -7.21 | <0.001 |
|  | Sampling Period × Treatment | -3.47 | 1.96 | -1.77 | 0.077 |
| Thatch depth | Intercept | 4.84 | 0.72 | 6.76 | <0.001 |
|  | Sampling Period | 0.49 | 0.34 | 1.44 | 0.151 |
|  | Treatment | -3.31 | 0.46 | -7.24 | <0.001 |
|  | Sampling Period × Treatment | -0.17 | 0.44 | -0.38 | 0.707 |
| Shrub cover | Intercept | -3.03 | 0.45 | -6.79 | <0.001 |
|  | Sampling Period | 1.24 | 0.22 | 5.52 | <0.001 |
|  | Treatment | -1.14 | 0.27 | -4.18 | <0.001 |
|  | Sampling Period × Treatment | -0.85 | 0.34 | -2.47 | 0.014 |

We recorded a total of 56 annual forb species in grazed and ungrazed prairies during the four survey years (S2 Table). The mean richness of native annual forbs declined between T1 and T2 by 2.8 species in grazed prairies and 0.1 species in ungrazed prairies, resulting in a significant interactive effect of time and grazing treatment on the richness of native annual forbs (Fig 2; Table 2). Native annual forb richness remained higher in grazed than ungrazed sites in T2, but the difference was much smaller than in T1. Native annual forb cover was greater in grazed than in ungrazed prairies during T1 (Fig 2; Table 2). During T2, mean native annual forb cover in grazed sites was more than twice as high in 2017 as compared to 2016, but was similar in both years in ungrazed sites.

**Fig 2:** Richness and cover of native annual forbs in grazed and ungrazed prairies in four survey years (n = 9 in 2000, 2016, n = 10 in 2001, 2017). Error bars indicate 1 SE.

**Table 2**. Fitted models for the effects of grazing and year on native annual forb richness and cover.

| **Model** | **Parameter** | **Estimate** | **Std. Error** | **z-value** | **p-value** |
| --- | --- | --- | --- | --- | --- |
| Native annual forb richness | Intercept | -1.10 | 0.37 | -3.01 | 0.003 |
|  | Sampling Period | -0.02 | 0.14 | -0.14 | 0.892 |
|  | Treatment | 1.09 | 0.16 | 7.01 | <0.001 |
|  | Sampling Period × Treatment | -0.39 | 0.16 | -2.40 | 0.017 |
| Native annual forb cover | Intercept | -0.41 | 0.50 | -0.82 | 0.411 |
|  | Sampling Period | 1.16 | 0.41 | 2.83 | 0.005 |
|  | Treatment | 1.60 | 0.45 | 3.57 | <0.001 |
|  | Sampling Period × Treatment | -0.73 | 0.48 | -1.51 | 0.132 |

The average SLA of species observed only in T1 (342.7 ± 43.0 mm^2^ g^-1^) was greater than those found in both (227.3 ± 18.0 mm^2^/g) periods or only in T2 (168.9 ± 17.0 mm^2^ g^-1^; n = 43 species, F = 3.3, p = 0.020). Most species observed in both sampling periods are classified as upland or facultative upland species (Fig 3). The proportion of species classified as ‘obligate wetland’, ‘facultative wetland’, or ‘facultative’ by the US Army Corps of Engineers declined from T1 (30%) to T2 (23%) and the proportion of facultative upland and upland species increased (Fig 3).

As I mentioned earlier, I do not believe these data analyses and results were subsequently discussed in the manuscript.

**Fig 3:** Wetland indicator status of native annual forbs observed in T1 (2000-2001) and in T2 (2016-2017).

Discussion

*Grassland structure*

The effects of grazing on vegetation structure were similar over time. In both sampling periods, grazed prairies had shorter standing living canopies and reduced thatch, as expected. The vegetation was significantly taller in both grazed and ungrazed plots in the second than in the first sampling period (T1:19.5 cm; T2: 29.0 cm), though the net difference in vegetation height between grazed and ungrazed plots was consistent over time (T1: Δ11.24 cm; T2: Δ14.6). In T2, mean vegetation height in grazed prairies approached but did not quite reach the T1 height of ungrazed prairies (Fig 1). The greater vegetation height in T2 could be attributed either to more precipitation in T2 than T1 (S2 Fig) or lower grazing pressure. Although we cannot conclusively distinguish between these two hypotheses, the fact that vegetation height was similar in both years of each sampling period, despite interannual differences in precipitation, suggests that grazing pressure was lower during T2.

Unfortunately, this sentence is not entirely true because cattle stocking rates were not analyzed. Please rephrase the sentence.

This sentence is speculative without interview data (e.g., structured or semi-structured interview). I recommend remove it.

I think is necessary to show any correlation data rather than citing only the figure.

I could not find where these results came from. Please cite the concerning table or figure.

I could not find where these results came from. Please cite the concerning table or figure.

do you mean "cover"?

Our resurvey supports the use of cattle grazing to reduce the rate of shrub encroachment into California coastal prairies. Many native coastal prairie species are adapted to disturbance regimes that prevent shrub encroachment, having evolved under grazing by Pleistocene megafauna [51] and later, frequent burning by Native peoples [52]. Shrub cover increased substantially more in ungrazed than grazed prairies over a 15-year period. The most common shrub species are mostly native, and their rate of spread are similar to those described from other coastal California sites [53, 54].

In which figure or table was presented this result? I understand that native vs exotic species were not compared.

*Native forb richness and cover*

Native annual forb species richness and cover continued to be greater in grazed than ungrazed prairies in the second sampling period, although the richness of grazed prairies had declined substantially over time. Our results are consistent with past research showing that shorter vegetation and higher levels of light at the soil surface increase native annual forb germination and growth when competition with exotic annual grasses is high [55, 56]. Reducing the canopy height and dominance of exotic annual grasses through grazing is a common grassland management technique, as exotic species often have faster growth rates and are competitively dominant to many native species in their early stages, reducing native species establishment [57-60]. Furthermore, the reduction of thatch by grazing can substantially enhance recruitment of native grassland species [61, 62].

The significant decline in the richness of native annual species we measured in grazed prairies complicates the interpretation of prior work showing a beneficial effect of grazing on native annual forbs in California [19, 20] and other Mediterranean grasslands [63-65]. Native annual forb species richness declined significantly more in grazed than ungrazed prairies, likely because ungrazed prairies had much lower richness to begin with, comprised of those species best able to compete with tall-statured exotic grasses. We cannot directly attribute the decline in native annual forb richness to changes in grazing regime or climate, but we use circumstantial evidence to explore the relative importance of these factors.

The significantly taller vegetation height in the second sampling period may explain part of the decline in native annual forb richness, given that many of the native annual forbs in California are low-stature species that benefit from greater light availability afforded by a shorter canopy [19, 20, 60]. However, vegetation height increased in both grazed and ungrazed plots, whereas native annual forb richness only declined in grazed sites. Thatch depth did not differ in grazed plots between the sampling periods and therefore likely did not cause the declines. There was significant treatment × time period interaction, as shrub cover increased substantially between time periods in ungrazed plots (~7 to 29%), but not in grazed plots (from ~1 to 4%). Thus, it seems unlikely that the primary cause for decline in native annual forb richness in grazed plots was local changes in vegetation structure.

Multiple lines of evidence support the hypothesis that climate and weather factors prior to the second survey led to the decline in native annual forb richness, though we note that we did not conduct manipulative experiments to directly link specific climate variables and species composition. California experienced a severe, 1-in-1200 year drought [33] between our sampling periods and the years immediately preceding our second sampling period were both exceptionally dry and warm (S2 Fig). Evidence from wetland indicator status and SLA, both of which are tied to plant water stress tolerance, suggest that increasing aridity, and therefore less plant-available water, may be an important contributor in the richness declines we measured. The native annual forb species absent in the second sampling period were disproportionately adapted to mesic environments according to their wetland indicator status (Fig 3). Moreover, the average SLA of plant species found only in T1 was significantly greater than for those found in T2 or both sampling periods, suggesting that the species that persisted are better adapted to drought. Likewise, other long-term grassland data in California [66] and experimental work by LaForgia and colleagues [67] show that native annual grassland forbs with traits associated with higher moisture conditions are more negatively affected by drought.

Importantly, grazing and precipitation often have interactive effects on native species richness and cover [40, 62, 68, 69]. Species richness generally increases under grazing in mesic and highly productive environments, but richness often declines under grazing in arid environments [11, 20, 70, 71] [but see 40]. Grazing during the extreme drought of 2012-2014 may have directly reduced native annual forb species richness, consistent with prior studies showing that grazing can reduce biodiversity during drought conditions [68, 69].

Overall native annual forb cover was greater in T2 than T1, and native annual forb cover was greater in grazed prairies than ungrazed prairies in three of four survey years (Fig 2). The greater native annual forb cover in ungrazed prairies in T2 as compared to T1 may be attributable to lagged drought-induced declines in exotic annual grasses [46, 72], slackening competitive pressure and allowing some species of native forbs to increase significantly in cover. Furthermore, the large spike in native annual forb cover in some grazed sites in 2017 as compared to 2016, suggests that the higher than typical precipitation in 2017 benefitted the native annual species that persisted through the drought but did little to restore native species richness, reinforcing findings from other sites that ‘drought plus deluge do not equal normal’ [35].

Our results show a pattern of declining native annual forb richness, but we are unable to determine whether the declines we observed are a short-term response to the 2012-2014 drought, changes in grazing, or a sign of longer-term trends. Heavy winter rainfall in 2017 did not result in increased site-level native annual forb species richness, which would be expected if high precipitation stimulated the persistent seedbanks typical of many species in this group, though a single high precipitation year may not be enough for all species to recover following severe drought. Prior work on the dynamics of annual forbs and annual grasses suggests that annual forbs persist alongside exotic annual grasses in part due to precipitation variability and drought [73, 74]. Additional data are necessary to determine whether the declines in richness we observed were due to local extinction or were only temporary responses to drought.

In conclusion, we find that grazed grasslands continue to have a greater richness of native annual forbs than ungrazed grasslands, but that the gap between grazed and ungrazed prairie diversity declined. Our study provides additional evidence that drought and rising temperatures affect community composition in managed grasslands globally [75, 76]. Finally, our study raises the question of whether species adapted to mesic conditions need alternative conservation strategies beyond *in situ* grazing in areas where aridity is increasing, such as assisted migration to climate refugia or *ex situ* collections.

**Acknowledgements**

We thank the UC Santa Cruz Reserve System, California State Parks, National Parks Service, Monterey Peninsula Regional Parks District, and private landowners for allowing us to access their land. We thank S. Glascock and M. Voce for their help in the field. We appreciate helpful feedback from M. Loik and S. Harrison on drafts of this paper.

**Data availability**

The data have been submitted to Dryad and the link will be added after acceptance.

**References**

1.	Asner GP, Elmore AJ, Olander LP, Martin RE, Harris AT. Grazing systems, ecosystem responses, and global change. Annual Review of Environment and Resources. 2004;29(1):261-99. doi: 10.1146/annurev.energy.29.062403.102142.

2.	Vellend M, Baeten L, Myers-Smith IH, Elmendorf SC, Beausejour R, Brown CD, et al. Global meta-analysis reveals no net change in local-scale plant biodiversity over time. Proc Natl Acad Sci U S A. 2013;110(48):19456-9. doi: 10.1073/pnas.1312779110. PubMed PMID: WOS:000327390400075.

3.	Dornelas M, Gotelli NJ, McGill B, Shimadzu H, Moyes F, Sievers C, et al. Assemblage time series reveal biodiversity change but not systematic loss. Science. 2014;344(6181):296-9. doi: 10.1126/science.1248484.

4.	Fleischner TL. Ecological costs of livestock grazing in western North America. Conserv Biol. 1994;8(3):629-44. doi: 10.1046/j.1523-1739.1994.08030629.x.

5.	Painter EL. Threats to the California flora: ungulate grazers and browsers. Madrono. 1995;42(2):180-8.

6.	Hilker T, Natsagdorj E, Waring RH, Lyapustin A, Wang Y. Satellite observed widespread decline in Mongolian grasslands largely due to overgrazing. Global Change Biology. 2014;20(2):418-28. doi: 10.1111/gcb.12365.

7.	Olff H, Ritchie ME. Effects of herbivores on grassland plant diversity. Trends Ecol Evol. 1998;13(7):261-5. doi: 10.1016/S0169-5347(98)01364-0.

8.	Rambo JL, Faeth SH. Effect of vertebrate grazing on plant and insect community structure. Conserv Biol. 1999;13(5):1047-54.

9.	de Bello F, Lepš J, Sebastià M-T. Variations in Species and functional plant diversity along climatic and grazing gradients. Ecography. 2006;29(6):801-10.

10.	Beck JJ, Hernández DL, Pasari JR, Zavaleta ES. Grazing maintains native plant diversity and promotes community stability in an annual grassland. Ecol Appl. 2015;25(5):1259-70. doi: 10.1890/14-1093.1.

11.	Lezama F, Baeza S, Altesor A, Cesa A, Chaneton EJ, Paruelo JM. Variation of grazing-induced vegetation changes across a large-scale productivity gradient. J Veg Sci. 2014;25(1):8-21. doi: 10.1111/jvs.12053.

12.	Montalvo J, Casado MA, Levassor C, Pineda FD. Species diversity patterns in Mediterranean grasslands. J Veg Sci. 1993;4(2):213-22. doi: 10.2307/3236107.

13.	Collins SL, Knapp AK, Briggs JM, Blair JM, Steinauer EM. Modulation of diversity by grazing and mowing in native tallgrass prairie. Science. 1998;280:745-7.

14.	Pykälä J. Effects of restoration with cattle grazing on plant species composition and richness of semi-natural grasslands. Biodiversity & Conservation. 2003;12(11):2211-26. doi: 10.1023/A:1024558617080.

15.	Van Auken OW. Shrub invasions of North American semiarid grasslands. Annu Rev Ecol Syst. 2000;3:197-215.

16.	Castro H, Freitas H. Above-ground biomass and productivity in the Montado: From herbaceous to shrub dominated communities. J Arid Environ. 2009;73(4-5):506-11. doi: 10.1016/j.jaridenv.2008.12.009.

17.	Bugalho MN, Caldeira MC, Pereira JS, Aronson J, Pausas JG. Mediterranean cork oak savannas require human use to sustain biodiversity and ecosystem services. Frontiers in Ecology and the Environment. 2011;9(5):278-86. doi: 10.1890/100084.

18.	Lyseng MP, Bork EW, Hewins DB, Alexander MJ, Carlyle CN, Chang SX, et al. Long-term grazing impacts on vegetation diversity, composition, and exotic species presence across an aridity gradient in northern temperate grasslands. Plant Ecol. 2018;219(6):649-63. doi: 10.1007/s11258-018-0824-4.

19.	Hayes GF, Holl KD. Cattle grazing impacts on annual forbs and vegetation composition of mesic grasslands in California. Conserv Biol. 2003;17(6):1694-702.

20.	Stahlheber KA, D’Antonio CM. Using livestock to manage plant composition: A meta-analysis of grazing in California Mediterranean grasslands. Biol Conserv. 2013;157:300-8. doi: 10.1016/j.biocon.2012.09.008.

21.	Borer ET, Seabloom EW, Gruner DS, Harpole WS, Hillebrand H, Lind EM, et al. Herbivores and nutrients control grassland plant diversity via light limitation. Nature. 2014;508:517-20. doi: 10.1038/nature13144.

22.	Fuhlendorf SD, Briske DD, Smeins FE. Herbaceous vegetation change in variable rangeland environments: The relative contribution of grazing and climatic variability. Appl Veg Sci. 2001;4(2):177-88. doi: 10.1111/j.1654-109X.2001.tb00486.x.

23.	Jonas JL, Buhl DA, Symstad AJ. Impacts of weather on long-term patterns of plant richness and diversity vary with location and management. Ecology. 2015;96(9):2417-32. doi: 10.1890/14-1989.1.

24.	Gornish ES, Ambrozio dos Santos P. Invasive species cover, soil type, and grazing interact to predict long-term grassland restoration success. Restor Ecol. 2016;24(2):222-9. doi: 10.1111/rec.12308.

25.	Bartolome JW, Allen-Diaz BH, Barry S, Ford LD, Hammond M, Hopkinson P, et al. Grazing for biodiversity in Californian Mediterranean grasslands. Rangelands. 2014;36(5):36-43.

26.	Ford LD, Hayes GF. Northern coastal scrub and coastal prairie. In: Barbour M, Keeler-Wolf T, Schoenherr AA, editors. Terrestrial Vegetation of California, 3rd edition. Berkeley: University of California Press; 2007. p. 180-207.

27.	Stromberg MR, Kephart P, Yadon V. Composition, invasibility, and diversity in coastal California grasslands. Madrono. 2001;48(4):236-52.

28.	Lulow ME. Restoration of California native grasses and clovers: the roles of clipping, broadleaf herbicide, and native grass density. Restor Ecol. 2008;16(4):584-93.

29.	Lee CC. Weather whiplash: Trends in rapid temperature changes in a warming climate. International Journal of Climatology. 2022:Forthcoming. doi: 10.1002/joc.7458.

30.	Swain DL, Langenbrunner B, Neelin JD, Hall A. Increasing precipitation volatility in twenty-first-century California. Nature Climate Change. 2018;8(5):427-33. doi: 10.1038/s41558-018-0140-y.

31.	Zamora-Reyes D, Black B, Trouet V. Enhanced winter, spring, and summer hydroclimate variability across California from 1940 to 2019. International Journal of Climatology. 2022:Forthcoming. doi: 10.1002/joc.7513.

32.	Williams AP, Seager R, Abatzoglou JT, Cook BI, Smerdon JE, Cook ER. Contribution of anthropogenic warming to California drought during 2012–2014. Geophysical Research Letters. 2015;42(16):6819-28. doi: 10.1002/2015GL064924.

33.	Griffin D, Anchukaitis KJ. How unusual is the 2012-2014 California drought? Geophysical Research Letters. 2014;41(24):9017-23. doi: 10.1002/2014GL062433.

34.	Copeland SM, Harrison SP, Latimer AM, Damschen EI, Eskelinen AM, Fernandez‐Going B, et al. Ecological effects of extreme drought on Californian herbaceous plant communities. Ecol Monogr. 2016;86:295-311.

35.	Harrison SP, LaForgia ML, Latimer AM. Climate-driven diversity change in annual grasslands: Drought plus deluge does not equal normal. Global Change Biology. 2018;24(4):1782-92. doi: 10.1111/gcb.14018.

36.	Westoby M, Falster DS, Moles AT, Vesk PA, Wright IJ. Plant ecological strategies: Some leading dimensions of variation between species. Annu Rev Ecol Syst. 2002;33(1):125-59. doi: 10.1146/annurev.ecolsys.33.010802.150452.

37.	Kimball S, Gremer JR, Angert AL, Huxman TE, Venable DL. Fitness and physiology in a variable environment. Oecologia. 2012;169(2):319-29. doi: 10.1007/s00442-011-2199-2.

38.	Fick SE, Hijmans RJ. WorldClim 2: new 1-km spatial resolution climate surfaces for global land areas. International Journal of Climatology. 2017;37(12):4302-15. doi: 10.1002/joc.5086.

39.	Hanley TA, Hanley KA. Food resource partitioning by sympatric ungulates on Great Basin rangeland. Rangeland Ecology & Management. 1982;35(2):152-8.

40.	Orr DA, Bui A, Klope M, McCullough IM, Lee M, Motta C, et al. Context-dependent effects of shifting large herbivore assemblages on plant structure and diversity. J Ecol. 2022:Forthcoming. doi: 10.1111/1365-2745.13871.

41.	Swain DL, Tsiang M, Haugen M, Singh D, Charland A, Rajaratnam B, et al. The extraordinary California drought of 2013/2014: Character, context, and the role of climate change. Bulletin of the American Meteorological Society. 2014;95(9):S3.

42.	Jepson Flora Project. Jepson eFlora. 2020;(7 July 2020):<https://ucjeps.berkeley.edu/eflora/>

43.	Kattge J, Diaz S, Lavorel S, Prentice C, Leadley P, Bonisch G, et al. TRY - a global database of plant traits. Global Change Biology. 2011;17(9):2905-35. doi: 10.1111/j.1365-2486.2011.02451.x.

44.	Sandel B, Corbin JD, Krupa M. Using plant functional traits to guide restoration: A case study in California coastal grassland. Ecosphere. 2011;2(2):art23. doi: 10.1890/ES10-00175.1.

45.	Tucker SS, Craine JM, Nippert JB. Physiological drought tolerance and the structuring of tallgrass prairie assemblages. Ecosphere. 2011;2(4):art48. doi: 10.1890/ES11-00023.1.

46.	LaForgia ML, Spasojevic MJ, Case EJ, Latimer AM, Harrison SP. Seed banks of native forbs, but not exotic grasses, increase during extreme drought. Ecology. 2018;99(4):896-903. doi: 10.1002/ecy.2160.

47.	Lichvar RW, Banks DL, Kirchner WN, Melvin NC. The National Wetland Plant List. Phytoneuron. 2016;30:1-17.

48.	R Core Team. R: A language and environment for statistical computing. 4.1.2 ("Bird hippie") ed. Vienna, Austria: R Foundation for Statistical Computing; 2021.

49.	Brooks ME, Kristensen K, Van Benthem KJ, Magnusson A, Berg CW, Nielsen A, et al. glmmTMB balances speed and flexibility among packages for zero-inflated generalized linear mixed modeling. The R journal. 2017;9(2):378-400. doi: 20.500.11850/242692.

50.	Hartig F. Diagnostics for hierarchical (multi-level / mixed) regression models. R package version 0.4.1 2021. Available from: <http://florianhartig.github.io/DHARMa/>.

51.	Wigand PE, Edwards S, Schiffman P. Pleistocene and pre-European grassland ecosystems. In: Stromberg MR, Corbin JD, D'Antonio CM, editors. California grasslands ecology and management University of California Press, Berkeley. Berkeley: University of California Press; 2007. p. 37-56.

52.	Anderson MK. Native American uses and management of California grasslands. In: Stromberg MR, Corbin JD, D'Antonio CM, editors. California Grasslands. Berkeley: University of California Press; 2007. p. 57-66.

53.	Russell WH, McBride JR. Landscape scale vegetation-type conversion and fire hazard in the San Francisco bay area open spaces. Landsc Urban Plann. 2003;64(4):201-8. doi: 10.1016/S0169-2046(02)00233-5.

54.	Zavaleta ES, Kettley LS. Ecosystem change along a woody invasion chronosequence in a California grassland. J Arid Environ. 2006;66(2):290-306. doi: 10.1016/j.jaridenv.2005.11.008.

55.	Coleman HM, Levine JM. Mechanisms underlying the impacts of exotic annual grasses in a coastal California meadow. Biol Invasions. 2007;9(1):65-71. doi: 10.1007/s10530-006-9008-6.

56.	Gillespie IG, Allen EB. Fire and competition in a southern California grassland: impacts on the rare forb *Erodium macrophyllum*. J Appl Ecol. 2004;41(4):643-52.

57.	Carlsen TM, Menke JW, Pavlik BM. Reducing competitive suppression of a rare annual forb by restoring native California perennial grasslands. Restor Ecol. 2000;8(1):18-29.

58.	Corbin JD, D'Antonio CM. Competition between native perennial and exotic annual grasses: implications for an historical invasion. Ecology. 2004;85(5):1273-83.

59.	Brandt AJ, Seabloom EW. Seed and establishment limitation contribute to long-term native forb declines in California grasslands. Ecology. 2012;93(6):1451-62. doi: 10.1890/11-0579.1.

60.	Marushia RG, Allen EB. Control of exotic annual grasses to restore native forbs in abandoned agricultural land. Restor Ecol. 2011;19(1):45-54.

61.	Molinari NA, D’Antonio CM. Where have all the wildflowers gone? The role of exotic grass thatch. Biol Invasions. 2020;22(3):957-68. doi: 10.1007/s10530-019-02135-1.

62.	Hernández E, Shaw EA, Aoyama L, Brambila A, Niederer C, Weiss SB, et al. Fire versus grazing as tools to restore serpentine grasslands under global change. Restor Ecol. 2021;29(S1):e13353. doi: 10.1111/rec.13353.

63.	Diaz S, Lavorel S, McIntyre S, Falczuk V, Casanoves F, Milchunas DG, et al. Plant trait responses to grazing - a global synthesis. Global Change Biology. 2007;13(2):313-41. doi: 10.1111/j.1365-2486.2006.01288.x. PubMed PMID: WOS:000244003700001.

64.	Noy-Meir I, Gutman M, Kaplan Y. Responses of Mediterranean grassland plants to grazing and protection. J Ecol. 1989;77:290-310.

65.	Osem Y, Perevolotsky A, Kigel J. Grazing effect on diversity of annual plant communities in a semi-arid rangeland: interactions with small-scale spatial and temporal variation in primary productivity. J Ecol. 2002;90(6):936-46. doi: 10.1046/j.1365-2745.2002.00730.x.

66.	Harrison S, Spasojevic MJ, Li D. Climate and plant community diversity in space and time. PNAS. 2020;117(9):4464-70. doi: 10.1073/pnas.1921724117.

67.	LaForgia ML, Harrison SP, Latimer AM. Invasive species interact with climatic variability to reduce success of natives. Ecology. 2020;101(6):e03022. doi: 10.1002/ecy.3022.

68.	Liang M, Chen J, Gornish ES, Bai X, Li Z, Liang C. Grazing effect on grasslands escalated by abnormal precipitations in Inner Mongolia. Ecology and Evolution. 2018;8(16):8187-96. doi: 10.1002/ece3.4331.

69.	Souther S, Loeser M, Crews TE, Sisk T. Drought exacerbates negative consequences of high-intensity cattle grazing in a semiarid grassland. Ecol Appl. 2020;30(3):e02048. doi: 10.1002/eap.2048.

70.	Schultz NL, Morgan JW, Lunt ID. Effects of grazing exclusion on plant species richness and phytomass accumulation vary across a regional productivity gradient: Grazing exclusion effects in grassy ecosystems. J Veg Sci. 2011;22(1):130-42. doi: 10.1111/j.1654-1103.2010.01235.x.

71.	Michaels J, Batzer E, Harrison S, Eviner VT. Grazing affects vegetation diversity and heterogeneity in California vernal pools. Ecology. 2021;102(4):e03295. doi: 10.1002/ecy.3295.

72.	Dudney J, Hallett LM, Larios L, Farrer EC, Spotswood EN, Stein C, et al. Lagging behind: have we overlooked previous-year rainfall effects in annual grasslands? J Ecol. 2017;105(2):484-95. doi: 10.1111/1365-2745.12671.

73.	Levine JM, Rees M. Effects of temporal variability on rare plant persistence in annual systems. Am Nat. 2004;164(3):350-63.

74.	Hallett LM, Shoemaker LG, White CT, Suding KN. Rainfall variability maintains grass-forb species coexistence. Ecol Lett. 2019;22(10):1658-67. doi: 10.1111/ele.13341.

75.	Gaitán JJ, Bran D, Oliva G, Maestre FT, Aguiar MR, Jobbágy E, et al. Plant species richness and shrub cover attenuate drought effects on ecosystem functioning across Patagonian rangelands. Biology Letters. 2014;10(10):20140673. doi: 10.1098/rsbl.2014.0673.

76.	Khishigbayar J, Fernández-Giménez ME, Angerer JP, Reid RS, Chantsallkham J, Baasandorj Y, et al. Mongolian rangelands at a tipping point? Biomass and cover are stable but composition shifts and richness declines after 20 years of grazing and increasing temperatures. J Arid Environ. 2015;115:100-12. doi: 10.1016/j.jaridenv.2015.01.007.

**Supporting information**

**S1 Table.** **Sites visited by sampling year**, ordered from North to South. Elevation extracted from the USGS National Map Elevation Point Query Service. Thirty-year mean temperatures and precipitation values extracted from the WorldClim version 2.1 climate model (Fick & Hijmans 2017).

**S2 Table.** **Native annual forb species observed in time periods 1 and 2.** Nomenclature follows Jepson Flora Project (2020).

**S1 Fig. Locations of sites sampled**. For site details see S1 Table. Data for state perimeters is from the U.S. Census Bureau.

**S2 Fig. Mean annual temperature (points) and precipitation (bars) for meteorological stations in the sampling region** (including Monterey, Santa Cruz, San Francisco, and Marin counties). The red dotted line shows the average temperature for the region over this period and the blue dashed line shows mean annual precipitation. Error bars indicate ±1 SE. The sampling years are shaded.
