# Supplementary material for: Native annual forbs decline in California coastal prairies over 15 years despite grazing
Source: PLoS One. 2022 Dec 6;17(12):e0278608. doi: 10.1371/journal.pone.0278608 (PMC9725146; doi:10.1371/journal.pone.0278608)
Supplement: Attachment — Submitted filename: Response to reviewers 2.docx [file pone.0278608.s006.docx]

**Editor and Journal Formatting Comments**

Based on the reviewers’ comments and my additional control, I am pleased to inform that your manuscript PONE-D-22-07590R1" Native annual forbs decline in California coastal prairies over 15 years despite grazing" can be accepted for publication in PLOS ONE after minor revision

*We have tried to thoroughly address all comments and hope that it is now acceptable for publication.*

Have the authors made all data underlying the findings in their manuscript fully available?
*The raw data are in the process of being published on Dryad and are available at* [*https://datadryad.org/stash/share/AcEqJVw6uwFA1I-at9CEnisDmxhtXQ8_uju2g_4Gjxo*](https://datadryad.org/stash/share/AcEqJVw6uwFA1I-at9CEnisDmxhtXQ8_uju2g_4Gjxo)*. They will be made publicly available upon acceptance.*

Please review your reference list to ensure that it is complete and correct.

*We have reviewed the reference list and updated the page and volume numbers of three references that were available early online at the time of our previous submission and are now officially published.*

While revising your submission, please upload your figure files to the Preflight Analysis and Conversion Engine (PACE) digital diagnostic tool, <https://pacev2.apexcovantage.com/>.

*We have checked to make sure that our figures are compliant using the PACE tool.*

**Reviewer Comments to the Author**

Reviewer #3: Dear Authors,
Overall the manuscript is a very nice attempt to explore the response of coastal prairies to changes in grazing management (grazed and ungrazed) in California. The paper is well written, and I think it is pleasant to read.

*We appreciate the supportive comments.*

However, some details can be improved, e.g., some unsupported sentences could be deleted, several concepts must be standardized, and minor changes in some figures should be made.

*We deleted the noted sentences, added details in response to comments, and made the recommended minor changes on the figures.*

Note that I do not find the "wetland indicator status" analyses helpful. But, it is up to you keeping or remove it.

*We have left the analysis on wetland indicator status in the paper. Our data clearly show that the number of annual forb species we observed in grazed grasslands declined between the two survey periods. We assert that the species lost were disproportionately those that are adapted to more mesic conditions, which we support with two lines of evidence – leaf area trait data and wetland indicator status. Several U.S. public agencies have worked together to develop a manual that categorizes plant species by their adaptation to mesic conditions in order to delineate wetlands. We think that the shift towards more arid, upland annual species is one piece of evidence that supports our assertation that aridification is at least partially driving the decline in annual forbs, so it is important to our paper, and we refer to it in the discussion. We removed some superfluous text in the data analysis section that the reviewer flagged where we referred to statistical analyses of these data that we didn’t report in the results.*

Beside this, I believe both supplementary figures should be in the manuscript's main text and not as an annex.

*We have moved both supplementary figures into the main text and have renumbered the figures accordingly.*

In the S1 Figure, please remove the words Longitude and Latitude; they are unnecessary.

*Figure revised as suggested. We also revised the multi-panel figures to include letters on each of the panels so we can refer to specific panels to support statements in the text.*

I enclose my specific comments in the Word file.
*Most of the comments in the Word file repeated comments listed above or were minor text edit suggestions, all of which we accepted. Additional comments that were not mentioned above include the following;*

- *The reviewer commented that we refer to the effect of grazing on exotic annual grasses despite not having measured exotic annual grass cover. We agree that we only report on vegetation height, thatch depth, and shrub cover not composition of the dominant species. Accordingly, we try to make that focus clear throughout the paper. However, most of the thatch and the tallest vegetation is non-native annual grasses, and it is impossible to talk about California grassland vegetation patterns without referring to the exotic annual grasses as part of the system. We draw on an extensive past literature to support our general statements in the introduction and discussion that exotic annual grasses dominate most California grasslands, commonly outcompete native species, and are negatively affect by grazing, particularly if they are tall stature grasses.*
- *The reviewer requested clarification on the type of cattle and the intensity of grazing. As we discussed in our response letter to the prior round of reviewer comments, we surveyed many sites over multiple years and there were a variety of grazing regimes across sites and years with respect to timing, intensity, and type of cattle. Unfortunately, we were not able to obtain detailed grazing records for every site for all the years. So, we have to generalize about the grazing regimes. Adding more detail than what is currently in the manuscript would suggest a higher level of precision of information than we have and would be misleading.*
- *The reviewer questioned our use of the word “structure” in the first sentence of the discussion and asked if we should use cover. We have clarified in parentheses that we use the term structure to refer to both vegetation height and cover.*
- *In the first paragraph of the discussion, the reviewer suggested that we do a correlation between precipitation and vegetation height at the two time periods. We do not think such a correlation analysis is defensible since we only have data from four years which are not distributed evenly over time (two years each at T1 and T2) whereas correlation analyses are used for variables that vary continuously. Moreover, other variables, such as grazing intensity, likely also varied between the two time periods, as we discuss. Therefore, we note that there are multiple plausible explanations for the change in vegetation height and weigh the evidence for the two most likely options, changes in grazing pressure and precipitation. We have modified the concluding sentence of this paragraph to note that evidence of reduced grazing pressure is anecdotal.*
- *In the second paragraph of the discussion the reviewer asked what evidence was presented to support the statement that “Many native coastal prairie species are adapted to disturbance regimes that prevent shrub encroachment, having evolved under grazing by Pleistocene megafauna and later, frequent burning by Native peoples.” We cite review articles that summarize a broad literature that supports each point. Given that these are historical patterns over thousands of years, no data were presented in the paper.*
- *The reviewer asked in the results and the second paragraph of the discussion for more detail on the percent cover of shrubs that were native. We have slightly reworded the text and added the percentage comprised by the top three shrub species. We didn’t feel that the space needed to add a separate figure or table for this information was warranted given that this isn’t a focal point of our manuscript.*

**Reviewer #4:** I have reviewed article 7590R1. I was not a reviewer of the original submission and consequently cannot comment on changes that were made to the previous version. Following my read of this version I have no serious concerns about the science contained in this work nor its interpretation. Some minor issues or suggestion for clarification include:

Line 24 – Specify ‘Livestock grazing’ grazing by native animals is typical of all grasslands etc.

*We reworded this sentence to clarify that nearly all grasslands have evolved with ungulate grazers, but humans have altered those grazing regimes enormously throughout the globe.*

Line 32-34 – There are just as many examples of livestock grazing encouraging woody plant encroachment as there are of it discouraging it. I would temper this statement and include the counterpoint that livestock can encourage encroachment.

*We have noted that overgrazing in shrublands can cause woody encroachment highlighting the importance of type of grazing regime and ecosystem in evaluating grazing impacts.*

Line 79 – Emphasize that these 10 sites are a subset of the original 26

*We added short phrase to clarify this point*

Line 84 – how many transects? I believe this question is answered later, but transect spacing is covered here. Perhaps a minor re-organization would put this connected material together in a single paragraph.

*We moved the sentence on the transect spacing and location to later in the methods where we describe the transects. We now only refer to characteristics of the full site at this point in the methods. We agree with the reviewer that this text needed reorganization and think the new ordering is more logical.*

Line 92 – Please clarify the stocking. The ‘1 cow per 2-4 ha’ corresponds to 1 cow-calf pair, or a single animal (i.e., does a cow-calf pair = 2 cows in this situation)?

*We have rephrased to say that this a cow-calf pair.*

Line 116 – Not necessary to say ‘are described below’. – *Deleted text as suggested.*

Line 118 – Please define ‘T1’. *We clearly defined T1 earlier in the methods but have re-clarified here that T1 refers to the “first” sampling period.*

Line 125-128 – Sentence describes two methods. Make separate sentences. *– Done*

Line 128-130 – Please elaborate on the method for quantifying cover. What technique was used?

*We have made minor wording changes to these sentences but think they are generally clear. We describe that “We calculated shrub cover as the number of woody shrubs pin intercepts as a percent of the 50 intercepts total along the transect” and that “we carefully searched for and measured the aerial cover (to the nearest cm^2^) of all native annual forbs present within a 1-m belt transect centered over the 50-m transect.”*

Line 135-136 = ‘When data … were available’ - *Corrected*

Line 141 – This is a really nice, super clear data analysis section! – *Thank you.*

Line 149 – Not familiar with the Tweedie – maybe provide a reference for this approach to your zero-inflated data. - *We have added a reference.*

Line 267-237 – I’m not understanding this argument that declines in native richness are greater in the ungrazed because they had low richness to begin with. Maybe expand on this thinking.

*We have expanded the text slightly to explain that if you start with fewer species then there are fewer species that can be “lost” over time. We recorded fewer annual forb species in ungrazed sites at T1 and by default those are species that are better able to compete with tall pasture grasses. Presumably some of the species that are weaker competitors and have more specific habitat requirements (e.g., moister soil conditions) were already absent from ungrazed sites at T1.*

Line 254 – You should do this again – its only gotten worse in CA since 2017…

*Unfortunately, this is true, and we agree that it would be interesting to do an additional survey now.*

Line 275 – better word choice for ‘slackening’? *Changed to reducing*

Line 298 – The thing I think this paper is missing – but not something you would address at this stage – is a community analysis because you have clearly observed a community shift toward more drought-tolerant species. Perhaps for the paper with 3 sampling periods in a few years! –

*We agree with these comments but think we need to sample a larger area for all guilds to be able to comment on species composition changes, since our current sampling focused on annual forbs.*
